# Immunohistochemical analysis of medullary breast carcinoma autoantigens in different histological types of breast carcinomas

**DOI:** 10.1186/1746-1596-7-161

**Published:** 2012-11-26

**Authors:** Olga Kostianets, Stepan Antoniuk, Valeriy Filonenko, Ramziya Kiyamova

**Affiliations:** 1 Department of Cell Signaling, Institute of Molecular Biology and Genetics, NAS of Ukraine, 150, Zabolotnogo str., Kyiv, Ukraine; 2 Educational and Scientific Centre “Institute of Biology”, Taras Shevchenko National University of Kyiv, 64, Volodymyrs’ka Str., Kyiv, Ukraine; 3 Dnipropetrovsk Clinical Oncological Center, Dnipropetrovsk, Ukraine

**Keywords:** Tumor-associated antigen, Expression pattern, Breast cancer

## Abstract

**Background:**

On the past decade a plethora of investigations were directed on identification of molecules involved in breast tumorogenesis, which could represent a powerful tool for monitoring, diagnostics and treatment of this disease. In current study we analyzed six previously identified medullary breast carcinoma autoantigens including LGALS3BP, RAD50, FAM50A, RBPJ, PABPC4, LRRFIP1 with cancer restricted serological profile in different histological types of breast cancer.

**Methods:**

Semi-quantitative immunohistochemical analysis of 20 tissue samples including medullary breast carcinoma, invasive ductal carcinoma, invasive lobular carcinoma and non-cancerous tissues obtained from patients with fibrocystic disease (each of five) was performed using specifically generated polyclonal antibodies. Differences in expression patterns were evaluated considering percent of positively stained cells, insensitivity of staining and subcellular localization in cells of all tissue samples.

**Results:**

All 6 antigens predominantly expressed in the most cells of all histological types of breast tumors and non-cancerous tissues with slight differences in intensity of staining and subcellular localization. The most significant differences in expression pattern were revealed for RAD50 and LGALS3BP in different histological types of breast cancer and for PABPC4 and FAM50A antigens in immune cells infiltrating breast tumors.

**Conclusions:**

This pilot study made possible to select 4 antigens LGALS3BP, RAD50, PABPC4, and FAM50A as promising candidates for more comprehensive research as potential molecular markers for breast cancer diagnostics and therapy.

**Virtual slides:**

The virtual slides’ for this article can be found here: http://www.diagnosticpathology.diagnomx.eu/vs/1860649350796892

## Background

Breast cancer is the most common female cancer in the world, with high mortality rate [1,2]. Identification of biomarkers for early detection and new therapeutic targets of breast cancer helps to continuously reduce the morbidity of this frequent pathology in women. This entails resolving the physiological, cellular and molecular processes underlying the complexity of breast tumor development and associated tumor heterogeneity [3]. In recent years efforts of many researchers are focused on search of new markers and molecules involved in breast tumorogenesis, which could be used in disease diagnostics, treatment and prognosis.

Development and application of proteomic technologies based on detection of autoantibodies to tumor antigens, including serological identification of antigens by recombinant expression cloning (SEREX) [4-6], serological proteome analysis (SEPRA) [7-9], multiple affinity protein profiling (MAPPing) [10] and high-density protein microarrays [11] have lead to identification of multiply biomarkers of breast cancer inducing autoantibody response. The characterization of tumor markers remains a major goal in both understanding the cellular mechanisms associated with tumorogenesis and in developing targets for the molecular therapy and diagnostics of cancer.

Protein tumor marker reflects consistent, biologically relevant changes in the tumor. It has been described that numerous autologous proteins of tumor cells, generally known as tumor-associated antigens (TAAs), can elicit humoral immune response in cancer patients as a result of their aberrant expression [12], alternative splicing of pre-messenger RNAs [13], point mutations [14], aberrant localization, folding [15], degradation [16] and/or post-translation alteration [17,18]. Many breast cancer antigens have been reported to be overexpressed at protein level in breast tumors; these include MUC1 [19], HER2/neu [20], P53 [21], HSP-27 [22,23], GIPC-1 [24], fibulin 1 [25] and cyclins B1 [26], D1 [27] and E [28]. Some tumor markers, for example, cyclin B1 also was found to change its location in cancer cells and localized predominantly in the cytosol [29], while normally it presents in the nucleus.

Our previous study was focused on identification of novel TAAs of medullary breast carcinoma (MBC), a relatively rare type of ductal carcinoma, which despite anaplastic features has favorable prognosis for patients [30-32]. High lyphocytic infiltration, indicating possible presence of specific antigen within tumor lesion is a distinctive feature of medullary breast carcinoma [30,33,34]. Using SEREX approach and serological plaque-spot assay we identified 41 potential antigens of medullary breast carcinoma, and showed that 18 of them had cancer restricted serological profile [35]. In this study protein expression pattern of a small subset of 6 from 18 (lectin, galactoside-binding, soluble, 3 binding protein (LGALS3BP); human RAD50 *S. cerevisiae* homolog (RAD50); family with sequence similarity 50, member A (FAM50A); poly(A) binding protein cytoplasmic 4 (PABPC4); recombination signal binding protein for immunoglobulin kappa J region (RBPJ) and leucine rich repeat (in FLII) interacting protein 1 (LRRFIP1)) antigens was investigated by immunohistochemical analysis of different histological types of breast cancer and non-cancerous breast tissues from patients with fibrocystic disease. Semi-quantitative analysis of positively stained cells, considering intensity of staining, and subcellular localization in normal, cancer and immune cells of lymphocytic infiltrate presented in some tumors using specifically generated polyclonal antibodies have been performed.

## Methods

### Clinicopathological data

Fresh breast cancer and non-cancerous breast tissue (NCT) samples were obtained from 15 female patients (39–75 years) with primary breast carcinoma and 5 patients with fibrocystic disease (37–50 years) correspondingly, who underwent surgery in the Dnipropetrovsk Clinical Oncological Center (Dnipropetrovsk, Ukraine) between 2008 and 2010. Surgical specimens were fixed with 10% buffered formalin, and paraffin embedded sections were stained with hematoxilin and eosin. All the tissue sample sections were reviewed to confirm the original diagnosis by an expert pathologist. The clinical and histopathological characteristics of these patients are shown in Table 1. Breast cancer cases included invasive ductal carcinoma (IDC) (n = 5), invasive lobular carcinoma (ILC) (n = 5), and medullary carcinoma (n = 5). Clinicopathological data were obtained from patient medical records and from the files kept at the Department of Pathohistology of Dnipropetrovsk Clinical Oncological Center. The study was approved by the Ethics Committee of the Institute of Molecular Biology and Genetics, NAS of Ukraine and informed consent was obtained from all patients.

**Table 1:** Characteristics of patients and their tissue samples

	**IDC (n = 5)**	**ILC (n = 5)**	**MBC (n = 5)**	**NCT (n = 5)**
Age (mean)	48.4	65.8	53	44.8
Age range	39-73	47-75	41-71	37-50
ER-positive	3	4	0	n/a
PR-positive	2	4	0	n/a
HER2-positive	3	2	0	n/a
Node positive	3	3	0	n/a
Ki-67 expression (range)	13-68%	8-29%	n/a	n/a

### Cloning, expression and purification of recombinant proteins

Specific cDNA of LGALS3BP, RAD50, FAM50A, RBPJ, PABPC4 and LRRFIP1 genes, isolated from MBC cDNA library Br502, was cloned into pGEX4T3 and/or pET28b expression vectors, containing glutathione-S-transferase- and 6His-tags respectively (Table 2). Expression of fused recombinant proteins was induced by 1 mM IPTG at 37°C for 4 h in E.coli BL21 (DE3) pLysE cells transformed by correspondent recombinant plasmids. Proteins were affinity purified using GST-sepharose and Ni-NTA-agarose according to manufacturers’ protocols. Some expressed recombinant proteins were present in the inclusion bodies of the pellet, which were washed before the purification procedure according X.A. Yang protocol [36]. Purity of proteins was analyzed by SDS-PAGE. All proteins were dialyzed against phosphate buffered saline (PBS), pH7.4, and then were used for immunization of mice. 

**Table 2:** Antigens used for generation of polyclonal antibodies

**Antigen**	**Full name**	**NCBI reference**	**DNA fragment, bp**	**Vector**	**MW of recombinant protein, kDa, (including tag)**
LGALS3BP	Homo sapiens lectin, galactoside-binding, soluble, 3 binding protein	NM_005567.2	1483-1686	pGEX4T3 (GST-tag)	35
RAD50	Homo sapiens RAD50 homolog (S. cerevisiae)	NM_005732.2	2552-3374	pET28b (6His-tag)	44
FAM50A	Homo sapiens family with sequence similarity 50, member A	NM_004699.1	76-1095	pGEX4T3 (GST-tag)	64
RBPJ	Homo sapiens recombination signal binding protein for immunoglobulin kappa J region	NM_203284.1	492-1850	pET28b (6His-tag)	50
PABCP4	Homo sapiens poly(A) binding protein, cytoplasmic 4 (inducible form)	NM_003819.2	927-3052	pET28b (6His-tag)	67
LRRFIP1	Homo sapiens leucine rich repeat (in FLII) interacting protein 1	NM_004735.2	417-1804	pET28b (6His-tag)	51

### Generation of polyclonal antibodies

Polyclonal antibodies against LGALS3BP, RAD50, FAM50A, RBPJ, PABPC4 and LRRFIP1 autoantigens were generated according the protocol described below. A primary dose of 20 mkg of each recombinant polypeptide was solubilized in Freund’s complete adjuvant (Sigma, Aldrich, Germany) and administered intraperitoneally in six- to 8-week-old female BALB/c mice four times at 2-week intervals. Then, immunized mice (with serum titer no less than 10^5^–10^6^) were boosted with 20 mkg of antigen in PBS by intraperitoneal injection. After 3 days of the booster injection, blood was collected from the mice, and the serum was separated.

Specificity of generated polyclonal antibodies was tested by Western blotting of recombinant proteins and confirmed by immunohystochemical analysis of MBC tumor, which was taken for generation of MBC cDNA library Br502 [37] with depleted against corresponding recombinant proteins polyclonal antibodies. Depletion of polyclonal antibody was performed as follows: antibodies diluted (1:10) in PBS were incubated overnight with correspondent recombinant proteins (10 mkg) transferred on PVDF membranes by standard blotting procedure. Decreasing of the intensity of immunohistochemical staining of Br502 tumor sections, when depleted antibody were used evidenced for specificity of generated polyclonal antibodies compared with undepleted ones.

### Immunohistochemical analysis

Immunohistochemical analysis of breast cancer samples with polyclonal Abs was performed according to standard protocol. Briefly, representative sections of breast tumors were prepared from parafin blocks. Endogenous peroxidase was quenched with H_2_O_2_ (3%) in 0.01% PBS. After blocking of non-specific binding with avidin-biotin blocking solution (Vector Laboratories, Burlingame, CA, USA), tissue sections were incubated overnight at 4°C with corresponded polyclonal antibodies (1:400). Then, sections were incubated with biotinylated secondary antibodies for 2 h at room temperature at 1:400 dilutions (goat anti-mouse biotinylated IgG, Sigma, Steinheim, Germany), followed by incubation with avidin-biotinperoxidase complex (Vector Laboratories, Burlingame, CA, USA) for 30 min at RT and developed with diaminobenzidine solution. Serum from non-immune mouse was used as negative control. Immunohistochemical staining of all breast cancer and non-cancerous tissue slides was performed during one experiment in equal conditions for each of antigens analyzed. Haematoxylin was used for counterstaining. Standard microscopy was performed using a Zeiss Universal microscope (Zeiss, Germany), and images were captured using digital Axiocam software.

Taking into account the fact that the expression of the studied antigens in normal and tumor breast tissues are poorly studied, we evaluated the staining intensity for each antigen comparing the slides of non-cancerous and cancerous breast tissue samples of different histological types, which were processed with correspondent polyclonal antibody under the same conditions. Tissue slides were analyzed using a four point semi-quantitative scale for nuclear and cytoplasmic optical staining intensity (graded as 3+ (strong), 2+ (moderate), 1+ (weak), 0 (no staining)) and for the percentage of positively stained cells (0 – no staining, 1 – <10% of the cells, 2 – 11 – 50% of the cells, 3 – >50% of the cells). Pathologist marked biologically representative areas, which contained a mean number of 150 cells (min 72 – max 180), percentage of positive cells was assessed as percent of cells exhibiting reactivity as average in 10 fields at high magnification (x400). The area of interest was selected randomly, excluding stromal cells and artifacts. Staining intensity and percent reactivity were recorded as mean observed in ten high power fields. The same slides were viewed again by each one of 3 month later, independently of the first vision.

## Results

In our previous studies, focused on identification of novel TAAs of medullary breast carcinoma, we found 41 antigens [37], 18 of which had cancer-restricted serological profile as was shown by phage based allogenic serological screening [35]. TAAs identified represent a diverse range of cellular proteins, some of which were shown to be implicated in cancer development, particularly lectin, galactoside-binding, soluble, 3 binding protein (LGALS3BP) [38], double-strand break repair factor RAD50 [39], nuclear protein with unknown function FAM50A [40], poly(A) binding protein cytoplasmic 4 (PABPC4) [41], mediator of Notch signaling RBPJ [42] and transcription factor LRRFIP1 [43]. To characterize their expression profile and subcellular localization in cancer cells of human breast carcinomas and cells of non-cancerous breast tissues immunohistochemical analysis using specific polyclonal antibodies has been performed. For generation of polyclonal antibodies cDNAs of LGALS3BP, RAD50, FAM50A, RBPJ, PABPC4 and LRRFIP1 antigens from MBC λ phage cDNA library were cloned in pGEX4T3 and pET28b plasmid vectors in the frame with GST- and His-tags correspondently for further affine purification (Table 2). All recombinant plasmids were expressed in bacteria and their affinity purified recombinant products were used for immunization of mice (Table 2). Specificity of generated antibodies was tested as described in Materials and Methods (data not shown).

**Figure 1:**
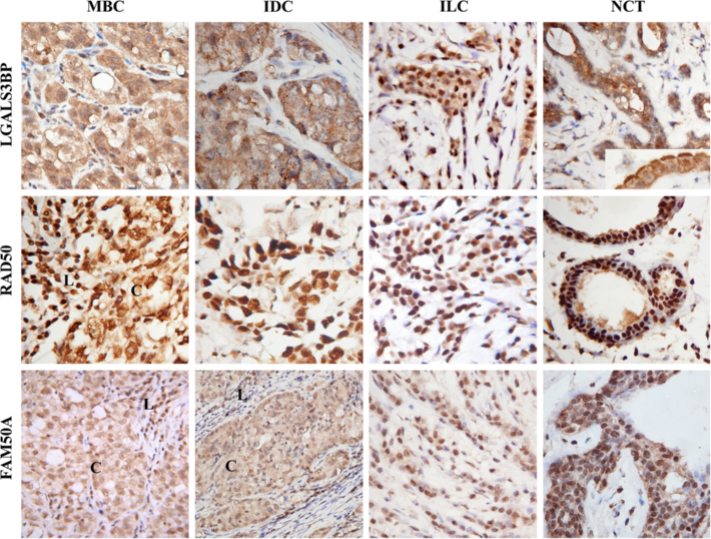
**Immunohystochemical staining reaction of LGALS3BP, RAD50, and FAM50A MBC-associated antigens in breast cancer and non-cancerous tissues.** Cancer cells of MBC and IDC demonstrated moderate to strong cytoplasmic and weak nuclear LGALS3BP staining, while in ILC samples weak cytoplasmic and moderate nuclear staining was observed. Strong positive LGALS3BP staining of cytoplasm and the apical part of normal epithelial cells was detected. Predominantly strong (3+) nuclear RAD50 staining in normal and cancer cells of MBC, IDC, and ILC was detected. Antigen FAM50A had moderate nuclear and weak cytoplasmic staining in NCT and tumor samples. C – cancer cells, L – lymphocytic infiltrate. Magnification 400x

Positive staining for LGALS3BP antigen was observed in more than 50% of all cells in tissue sections of the most breast tumors types and non-cancerous breast tissues (Figure 1), (Table 3). Tumor cells of IDCs and MBCs showed weak nuclear (1+) and moderate (2+) to strong (3+) cytoplasmic staining, while all ILCs showed opposite expression pattern with weak (1+) cytoplasmic and moderate (2+) nuclear staining (Table 4). Notably, strong LGALS3BP staining was observed at the apical part of normal epithelial cells of the ducts and lobules in non-cancerous tissues (Figure 1) and in some ILCs cases.

**Table 3:** Descriptive data of 6 antigens expression in tissue samples

**Antigen**	**Tissue sample**	**Positive cells (%)**											
		**Normal cells**				**Cancer cells**				**Immune cells**			
		**0**	**1**	**2**	**3**	**0**	**1**	**2**	**3**	**0**	**1**	**2**	**3**
LGALS3BP	MBC	n/a	n/a	n/a	n/a	-	-	1/5	4/5	-	-	1/5	4/5
	IDC	n/a	n/a	n/a	n/a	-	-	1/5^*^	4/5	-	-	1/1^*^	-
	ILC	n/a	n/a	n/a	n/a	-	-	-	5/5	-	-	-	-
	NCT	-	-	-	5/5	-	-	-	-	-	-	-	-
RAD50	MBC	n/a	n/a	n/a	n/a	-	-	-	5/5	-	-	-	5/5
	IDC	n/a	n/a	n/a	n/a	-	-	1/5^*^	4/5	-	-	-	1/1^*^
	ILC	n/a	n/a	n/a	n/a	-	-	-	5/5	-	-	-	-
	NCT	-	-	-	5/5	-	-	-	-	-	-	-	-
FAM50A	MBC	n/a	n/a	n/a	n/a	-	-	-	5/5	-	-	1/5	4/5
	IDC	n/a	n/a	n/a	n/a	-	-	1/5^*^	4/5	-	-	-	1/1^*^
	ILC	n/a	n/a	n/a	n/a	-	-	2/5	3/5	-	-	-	-
	NCT	-	-	-	5/5	-	-	-	-	-	-	-	-
RBPJ	MBC	n/a	n/a	n/a	n/a	-	-	-	5/5	-	-	-	5/5
	IDC	n/a	n/a	n/a	n/a	-	-	1/5^*^	4/5	-	-	-	1/1^*^
	ILC	n/a	n/a	n/a	n/a	-	-	-	5/5	-	-	-	-
	NCT	-	-	-	5/5	-	-	-	-	-	-	-	-
PABPC4	MBC	n/a	n/a	n/a	n/a	-	-	-	5/5	-	-	1/5	4/57^**^
	IDC	n/a	n/a	n/a	n/a	-	-	1/5^*^	4/5	-	-	-	1/1^*^
	ILC	n/a	n/a	n/a	n/a	-	-	-	5/5	-	-	-	-
	NCT	-	-	-	5/5	-	-	-	-	-	-	-	-
LRRFIP1	MBC	n/a	n/a	n/a	n/a	-	-	-	5/5	-	-	-	5/5
	IDC	n/a	n/a	n/a	n/a	-	-	1/5^*^	4/5	-	-	1/1^*^	-
	ILC	n/a	n/a	n/a	n/a	-	-	-	5/5	-	-	-	-
	NCT	-	-	-	5/5	-	-	-	-	-	-	-	-

MBC – medullary breast carcinoma, IDC-invasive ductal carcinoma, ILC – invasive lobular carcinoma, NCT – non-cancerous tissue; n/a - normal cells were not available within tumor sample; 0 - no staining, 1 - <10% of the positively stained cells, 2 - 10-50% of the positively stained cells, 3 > 50% of the positively stained cells; - one case of IDC which was heavily infiltrated by lymphocytes; ** - PABPC4 positive staining was observed in 50-70% of immune cells.

**Table 4:** Descriptive data of 6 antigens intracellular distribution and insensitivity of staining in tissue samples^*^

			**Antigens**					
**Case**	**Tissue sample**	**Age**	**LGALS3BP**	**RAD50**	**FAM50A**	**RBPJ**	**PABPC4**	**LRRFIP1**
1	MBC	42	N1+, C2+	N3+	N1+, C1+	N2+, C1+	C3+	N2+, C2+
2	MBC	40	N1+, C3+	N2+	N2+, C2+	N2+, C1+	C3+	N2+, C2+
3	MBC	46	N1+, C3+	N3+	N1+, C1+	N2+, C1+	C2+	N3+, C2+
4	MBC	70	N2+, C2+	N3+, C1+	N2+, C1+	N3+, C1+	C3+	N3+, C1+
5	MBC	57	N1+, C3+	N3+	N2+, C1+	N3+, C1+	C3+	N2+, C2+
6	IDC	66	N1+, C3+	N3+	N2+, C1+	N2+, C1+	C3+	N3+, С1+
7	IDC	38	N1+, C2+	N2+	N2+, C2+	N2+, C1+	C2+	N3+, С1+
8	IDC	68	N1+, C2+	N3+	N2+, C1+	N3+, C2+	C3+	N2+, С1+
9	IDC	72	N1+, C2+	N3+	N2+, C1+	N2+, C1+	C3+	N3+, С1+
10	IDC	63	N1+, C3+	N3+	N2+, C1+	N3+, C1+	C2+	N2+, С1+
11	ILC	66	N2+, C1+	N2+	N2+, C1+	N2+, C1+	C3+	N2+
12	ILC	38	N2+, C1+	N3+	N2+, C1+	N2+	C2+	N3+, C1+
13	ILC	68	N2+, C1+	N2+	N2+, C1+	N2+	C3+	N3+, C1+
14	ILC	72	N2+, C1+	N2+	N2+, C1+	N2+, C1+	C2+	N2+
15	ILC	63	N2+, C1+	N2+	N2+, C1+	N2+	C3+	N3+, C1+
16	NCT	43	N2+, С1+	N3+	N2+, C1+	N1+, C1+	C3+	N2+, C1+
17	NCT	50	N1+, C2+	N3+, C1+	N3+, C1+	N2+, C1+	C3+	N3+
18	NCT	46	N1+, C2+	N3+	N2+, C1+	N1+, C1+	C3+	N2+, C1+
19	NCT	48	N2+, С1+	N3+	N2+, C1+	N2+	C2+	N3+, C1+
20	NCT	37	N1+, C1+	N3+	N2+, C1+	N2+	C2+	N3+

MBC – medullary breast carcinoma, IDC – invasive ductal carcinoma, ILC – invasive lobular carcinoma, NCT – non-cancerous tissue. N – nuclear staining, C – cytoplasmic staining; ^*^ staining intensity was graded as 3+ (strong), 2+ (moderate), 1+ (weak).

All 15 breast cancer and 5 non-cancerous tissue samples were positive for the DNA reparation factor RAD50 (Figure 1), (Table 3). We observed moderate (2+) to strong (3+) nuclear RAD50 staining with no cytoplasmic staining apparent in normal and cancer cells in the most cases. However, in 4 of 5 ILCs cases decreasing of the intensity of nuclear staining (moderate (2+)) was detected.

Analysis of FAM50A protein expression revealed that more than 50% of cells were positively stained in cancer and non-cancerous tissue sets (Figure 1), (Table 3). FAM50A protein predominantly was distributed in nucleus and cytoplasm of cancer cells of all tumor samples with preferentially moderate (2+) and weak (1+) positive staining respectively (Table 4).

**Figure 2:**
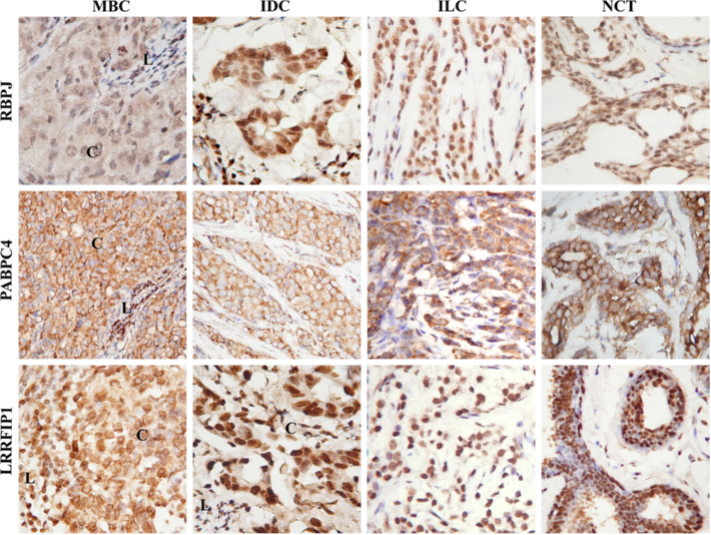
**Immunohystochemical staining reaction of RBPJ, PABPC4, and LRRFIP1 MBC-associated antigens in breast cancer and non-cancerous tissues.** Antigen RBPJ had moderate nuclear and weak cytoplasmic staining of normal and cancer cells of NCT, MBC, and IDC, except for ILC samples which showed only moderate (2+) nuclear staining. Moderate to strong positive PABPC4 staining was detected in cytoplasm of normal and cancer cells of non-cancerous and cancer tissue samples. Cancer cells of IDC and ILC, and normal cells of non-cancerous tissues had moderate to strong nuclear and weak cytoplasmic LRRFIP1 staining, but in MBC moderate cytoplasmic LRRFIP1 staining was observed. C – cancer cells, L – lymphocytic infiltrate. Magnification 400x

RBPJ staining was indicated in the most of cancer and normal cells (Figure 2), (Table 3). Moderate (2+) to strong (3+) nuclear and weak (1+) cytoplasmic staining (Table 4) was detected in MBC and IDC tissue samples. In ILC and NCT tissues samples predominantly moderate (2+) nuclear and additional weak (1+) cytoplasmic staining in some cases was detected.

Moderate (2+) to strong (3+) PABPC4 positive staining was exclusively observed in cytoplasm of normal and cancer cells of non-cancerous and cancer tissue samples with no relation to histological type (Figure 2), (Tables 3 and 4).

**Figure 3:**
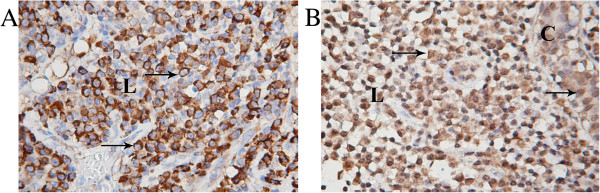
**Immunohistochemical staining of PABPC4 (A) and FAM50A (B) antigens in cells of lymphocytic infiltrate of medullary breast carcinoma.** Both antigens showed more intensive staining of immune cells compared with cancer cells. Positive cytoplasmic PABPC4 staining was detected only in a part of immune cells. FAM50A showed preferentially nuclear staining in immune cells. Positively stained cells are indicated by arrows. C – cancer cells, L – lymphocytic infiltrate. Magnification 400x

LRRFIP1 nuclear and cytoplasmic expression was indicated in more than 50% of all tissue samples (Figure 2), (Table 3). This protein was located in nucleus and cytoplasm of both cancerous and non-cancerous tissue sets with moderate (2+) to strong (3+) nuclear and weak (1+) cytoplasmic staining, however in MBC tissues moderate (2+) cytoplasmic LRRFIP1 staining was observed (Figure 2). According to the literature data some of the analyzed proteins could be involved in modulation of immune response [41,42,44-46]. Taking this fact into account we performed immunohistochemical analysis of tumor regions of all MBC and 1 case of IDC highly infiltrated by lypmphocytes, with correspondent polyclonal antibodies. We have shown that all proteins expressed in the most of immune cells of lymphocytic infiltrate (up to 90%) (Table 3), with exception for PABPC4 which positive staining was observed only in 50–70% of immune cells (Figure 3). Moreover, PABPC4 showed more intensive cytoplasmic staining of immune cells compared with cancer and normal cells (Figure 3), as well as nuclear antigen with unknown function FAM50A. More intensive compared with cancer and normal cells FAM50A positive staining was detected in the nucleus of immune cells (Figure 3). 

## Discussion

The identification of breast cancer markers has been the focus of extensive research in past decades, there still an urgent need for good cancer biomarkers remains. Moreover, characterization of these proteins and identification of the alterations that distinguish cancer cells from normal ones will help to understand the molecular mechanisms of cancerogenesis. In previous study we identified by SEREX-approach 41 novel medullary breast carcinoma autoantigens, 18 of which had cancer-related serological profile [35]. During this study expression pattern of 6 from 18 potential MBC-derived antigens were studied by immunohistochemistry in tissue samples obtained from patients with different histological types of breast cancer and fibrocystic disease using specifically generated polyclonal antibodies. Medullary breast carcinoma, lobular and ductal invasive breast carcinomas’ tissue samples were taken for immunohistochemical investigation of expression patterns of above mentioned proteins. It’s known that lobular and ductal carcinomas derive both from the breast terminal duct lobular units [47] and the terminology “ductal” and “lobular” is still being used for historical reasons and to date there is no evidence to suggest that these tumors arise from ductal or lobular epithelial cells. Thus, the differences in their morphology are likely to reflect the different mechanisms of carcinogenesis rather than the anatomical origin of the lesions [48]. For example, it was demonstrated that 5.8% of the transcriptionally regulated genes were differentially expressed in ILCs compared to grade- and molecular subtype-matched IDCs [49]. Identification and characterization of molecular targets specific for each type of breast cancer will help to design more precise therapeutic and diagnostic approaches for curing of this type of malignancy.

During this preliminary study, we investigated expression pattern of 6 MBC autoantigens, including secretory protein LGALS3BP, 2 nuclear proteins (RAD50, FAM50A), one cytoplasmic protein (PABPC4) and 2 tratscription factors (RBPJ, LRRFIP1), in different histological types of breast cancer and non-cancerous tissue samples by immunohistochemical analysis. Despite the fact that automated measurement using digital slide scanners and computer-aid methods are increasingly used for immunohystochemical analysis, during this study we used visual semi-quantitative evaluation of MBC antigens expression pattern by expert pathologist because high correlation between these two approaches was recently shown [50-52].

It was shown that all analyzed proteins expressed in the most cells of non-cancerous and cancer tissue samples. However distinct expression pattern was revealed for some antigens in breast carcinomas of different histological types.

LGALS3BP (also known as MAC-2BP and 90K protein) was originally identified as a tumor-associated antigen in the culture supernatant of human breast cancer cells [53]. Early works focused on study of LGALS3BP showed its expression in more than 80% of breast cancer tissues, but not in non-cancerous normal mammary gland surrounding the cancer cells [53]. Immunohistochemical analysis of LGALS3BP expression in several tumors, including breast carcinomas, revealed LGALS3BP positive staining predominantly in cytoplasm [54]. During our study, we also, observed moderate to strong LGALS3BP positive staining in cytoplasm and weak nuclear staining of IDC and MBC cells. Interestingly, that in ILC tumors we detected opposite pattern of LGALS3BP expression with moderate nuclear and weak cytoplasmic staining. Moreover, during this study we observed an explicit apical membrane staining of LGALS3BP in some of epithelial cells in breast tissues of patients with fibrocystic disease and epithelial cells lining the normal ducts in ILCs, contrary to mentioned above literature data [53]. This strong apical membrane staining of LGALS3BP may partly be explained by its ability to be secreted by different types of cells including epithelial cells [55-57].

RAD50 is a highly conserved DNA double-strand break repair factor [39]. Its aberrantly reduced protein expression was reported in 3% of breast tumors (predominantly ER/PR/ERBB2 triple-negative and higher-grade familial breast tumors) [57]. In our investigation we also showed strong RAD50 nuclear staining of non-cancerous and cancer cells. However, in 4 from 5 ILC cases we observed rather moderate than strong RAD50 positive staining in the nucleus of cancer cells. Since, down-regulation of some genes including RAD50 was shown in ILCs [49]; our results partially confirmed the data about reduced RAD50 expression in ILC tumors. However, this fact should to be further investigated on a large number of samples.

FAM50A (also known as XAP5) is a poorly studied protein with unknown function having signal for nuclear localization [40]. Here, we describe for the first time its subcellular localization and expression pattern in cancer and non-cancerous breast tissues. According to our findings FAM50A protein is expressed both in nucleus and cytoplasm in the most cells of cancer and non-cancerous breast tissues without any significant differences. However we found stronger FAM50A nuclear staining in immunocytes compared with cancer and non-cancerous breast cells.

PABPC4 (or inducible PABP (iPABP)) a homolog of PABP, have critical roles in RNA processing beyond simply binding to poly(A) sequences [41]. According to the literature data PABPC4 is a diffusely cytoplasmic protein that can be localized to stress granules [58]; its expression is induced upon stimulation of peripheral T cells [41]. The fact that induction of PABPC4 coincides with the induction of lymphokine mRNA in activated T cells suggests that perhaps iPABP is necessary for regulation of stability of labile mRNA species [41]. In our study, we observed moderate to strong PABPC4 positive staining in cytoplasm in the most of cancer and normal cells of all tissue samples and in part of immunocytes. One may be speculated that PABPC4 expression can be restricted to activated T cells at least in MBC tumors, and possibly contributes to a more effective immune response that can be associated with good prognosis for patients with this tumor type. However, to confirm this assumption additional study has to be performed.

RBPJ is a primary transcription mediator of canonical Notch signaling [59], which participates in cell fate determination, and is involved in the regulation of tumor behavior in multiple decisions [60]. It has been shown previously, that RBPJ protein can be detected in the nucleus as well as in the cytoplasm and it subcellular distribution changes in defined physiological contexts [61-64].

In all cancer and non-cancerous cases predominantly moderate nuclear RBPJ and weak cytoplasmic staining was detected, however in some MBC and IDC cases strong nuclear staining was revealed. These differences may be associated with histological and molecular features of these tumor types.

LRRFIP1 is ubiquitously expressed and found in the nuclear and cytoplasmic compartments [65]. This protein acts as transcriptional repressor of some genes [45,46,66] and indirectly throught interaction with Flightless 1 protein involved in actin severing, capping and cytoskeletal rearrangement [67]. Notably, LRRFIP1 can be mutated in breast tumors and soft tissue sarcomas [68], and may be involved in the regulation of cell growth [43]. We observed strong nuclear and weak cytoplasmic distribution of LRRFIP1 in cancer and normal cells except for MBC tumors, which showed moderate cytoplasmic staining in addition to strong nuclear staining.

Thus, during this study we described for the first time expression patterns of 6 potential MBC-associated antigens, including LGALS3BP, RAD50, FAM50A, RBPJ, PABPC4, LRRFIP1 in different histological types of breast carcinomas and non-cancerous breast tissues by immunohistochemical analysis. Our data indicate that all 6 antigens predominantly expressed in the most cells of all histological types of breast tumors, non-cancerous tissues, and immunocytes with slight differences in intensity of staining and subcellular localization. The most significant differences were observed for RAD50 and LGALS3BP antigens in different histological types of breast carcinomas. During this study we described for the first time expression pattern of poorly studied antigen FAM50A and observed its more intensive nuclear staining in the immune cells compared with cancer and non-cancerous cells. Additionally increased expression of other antigen PABPC4 was observed in restricted number of immune cells infiltrating breast tumors. These observations possibly reflect molecular changes that occur during breast carcinogenesis in different histological types of breast cancer and indicate that RAD50 and LGALS3BP antigens are promising candidates for more comprehensive research as potential molecular markers for breast cancer diagnostics and therapy. Moreover, antigens FAM50A and PABPC4 are intriguing targets for investigation of the features of the immune response in patients with highly infiltrating breast tumors including MBC, which despite anaplastic features has favorable prognosis.

## Conclusions

In this study we determined for the first time expression patterns of 6 potential MBC-associated antigens, including LGALS3BP, RAD50, FAM50A, RBPJ, PABPC4, and LRRFIP1, in non-cancerous cells of the breast, cancer and immune cells of different histological types of breast carcinomas by immunohistochemical analysis. The most significant differences in expression pattern were revealed for RAD50 and LGALS3BP in cancer cells of different histological types of breast cancer and for PABPC4 and FAM50A antigens in immune cells infiltrating breast tumors. This work is a pilot study, which made possible to select 4 potential breast cancer markers (FAM50, PABC4, RAD50, LGALS3BP) for further studies with larger cohorts of patients with different histological types of breast cancer using automated measurement systems for immunohistochemical analysis. Data obtained can contribute to investigations in the field of breast cancer genesis, diagnostics and therapy, and may be of interest for a wide range of researchers including those who already focused on precise study of indicated proteins.

## Abbreviations

SEREX: Serological identification of antigens by recombinant expression cloning; SEPRA: Serological proteome analysis; MAPPing: Multiple affinity protein profiling; TAAs: Tumor-associated antigens; MBC: Medullary breast carcinoma; NCT: Non-cancerous breast tissue; IDC: Invasive ductal carcinoma; ILC: Invasive lobular carcinoma; LGALS3BP: Lectin, galactoside-binding, soluble, 3 binding protein; RAD50: Human RAD50 *S. cerevisiae* homolog; PABPC4: Poly(A) binding protein, cytoplasmic 4; FAM50A: Family with sequence similarity 50, member A; RBPJ: Recombination signal binding protein for immunoglobulin kappa J region; LRRFIP1: Transcription factor leucine rich repeat (in FLII) interacting protein 1.

## Competing interests

The authors declare that they have no competing interests.

## Authors’ contributions

OK and SA carried out the experiments and analysis of results obtained. OK, VF, RG participated in the design of the study, analysis of obtained results and drafted the manuscript. All authors have read and approved the final manuscript.
